# Century-long Taylor-Quinney interpretation of plasticity-induced heating reexamined

**DOI:** 10.1038/s41598-019-45533-0

**Published:** 2019-06-24

**Authors:** Aleksander Zubelewicz

**Affiliations:** Civil Engineering Department, University of New Mexico, Albuquerque, NM USA

**Keywords:** Condensed-matter physics, Materials science, Mechanical engineering

## Abstract

In thermomechanics, the Taylor–Quinney coefficient specifies fraction of plastic work converted to heat. We challenge the nearly century-long interpretation. We postulate that some fraction of energy delivered to the plastically deformed material is responsible for readjustments of deformation pathways making the plastic flow a kinematically admissible process. The rerouting triggers mesoscale dynamic excitations and activates plasticity-induced heat. Another part of the energy is stored in lattice, while the rest of it contributes to the development of dislocation structures. According to this interpretation, plastic work is not converted to heat, but increases probability of the microstructural adaptiveness and, in this manner, contributes to configurational entropy of the system.

## Introduction

**Figure 1:**
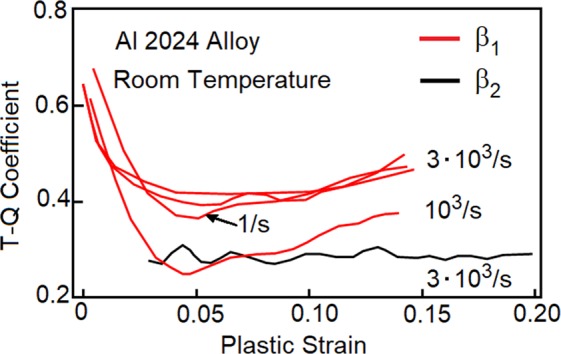
Taylor-Quinney coefficient for Al 2024 alloys is presented as a function of plastic strain. The instantaneous coefficient *β*_1_ is marked in red and *β*_2_ is shown in black. The data is gathered from refs^6–9^.

In nineteenth century, experiments conducted by Tresca showed that metals subjected to large plastic deformation experience noticeable heating. He estimated that 73% to 94% of plastic work is converted to heat. Many years later, Taylor and Quinney calculated efficiency of the process and arrived to the conclusion that about 90% of plastic work is turned into heat. They determined that a plastically deformed metal stores small portion of plastic work, thereby raises its internal energy^1–4^. More recently, the estimates are significantly revised. The amount of cold work is found to be much larger than initially thought, and it depends on plastic strain, strain rate and type of loading. Unfortunately, the measurements are endowed with a significant scatter^5–7^ and convey unclear message about the phenomena. It is reported that the strongest production of heat occurs at the initial stage of plastic flow. Further increase of plastic deformation slows down the process, and then, the rate increases again at even more advanced deformation. Experiments conducted on aluminum alloy Al 2024 properly capture the trends^6–9^, Fig. 1. The process is often monitored in terms of a coefficient, which specifies the ratio of heat rate taken over plastic power ${\beta }_{1}=\rho \,{C}_{p}\,\dot{T}/{\dot{W}}^{p}$. Conversely, the rate of cold work is defined as $(1-\,{\beta }_{1}){\dot{W}}^{p}$. We emphasize that similar behaviors are observed in CPTi, Cu, steels, and other materials. Frequently, the plasticity-generated heat is estimated with the use of integral coefficient *β*_2_ = *ρC*_*p*_*T*/*W*^*p*^. The coefficient is evaluated for several materials^7^ subjected to strain rates in the Kolsky-bar regime. The average values of *β*_2_ are found to vary between 0.2 and 0.9. Thus, there is little consistency between different materials. Several attempts have been made to justify the Taylor-Quinney interpretation. For example, the concept is used as the departure point in a thermodynamics description presented in ref.^9^. Also, it is worth noting that the cold work storage is analyzed in discrete dislocation plasticity method simulations^10^. Most often though, heuristic (curve fitting) estimates are proposed.

We challenge the Taylor-Quinney interpretation. The argument runs as follows. Let’s consider a material subjected to plastic deformation. As reported^11^, the process is chaotic and requires dislocation overcoming flow-impeding obstacles. Consequently, the deformation pathways are continuously rerouted and the zigzagging triggers small-scale excitations. The externally supplied energy is partly stored in the lattice, is utilized to do plastic work, while the excitations consume the remaining portion of the energy. In this scenario, the macroscopically measured plastic work ${W}^{p}=\int {\boldsymbol{\sigma }}:d{{\boldsymbol{H}}}^{pe}$ is not converted to heat and, instead, *W*^*p*^ increases probability of occurrence of kinematically admissible dislocation structures making the plastic flow a thermodynamically optimal process. In here, stress and plastic strain are ***σ*** and ***H***^*pe*^, respectively. In essence, we suggest that plastic work increases configurational entropy of the system^11,12^, while plasticity-induced heating quantifies efficiency of the process.

## Results

The study is based on the fundamental principle that internal energy must exceed the existing energy barriers in order for plastic deformation to be initiated, where the word “exceed” is of relevance. Secondly, kinematical adaptiveness of dislocation structures assures that the plastic flow is a thermodynamically optimal process. The path rearrangements consume some portion of energy delivered to the material. Thus, the crystallographic structure, crystal size, grain-to-grain orientations, dislocation structures and other defects; all the factors affect efficiency of the process. At quasi-static conditions, the rerouting-induced excitations are quickly dissipated. In the extremes of dynamics, the rate of energy delivered to the material exceeds the rate at which the material accommodates the energy. The imbalance pushes the material even further from the near equilibrium state of behavior.

### Stress perturbations

**Figure 2:**
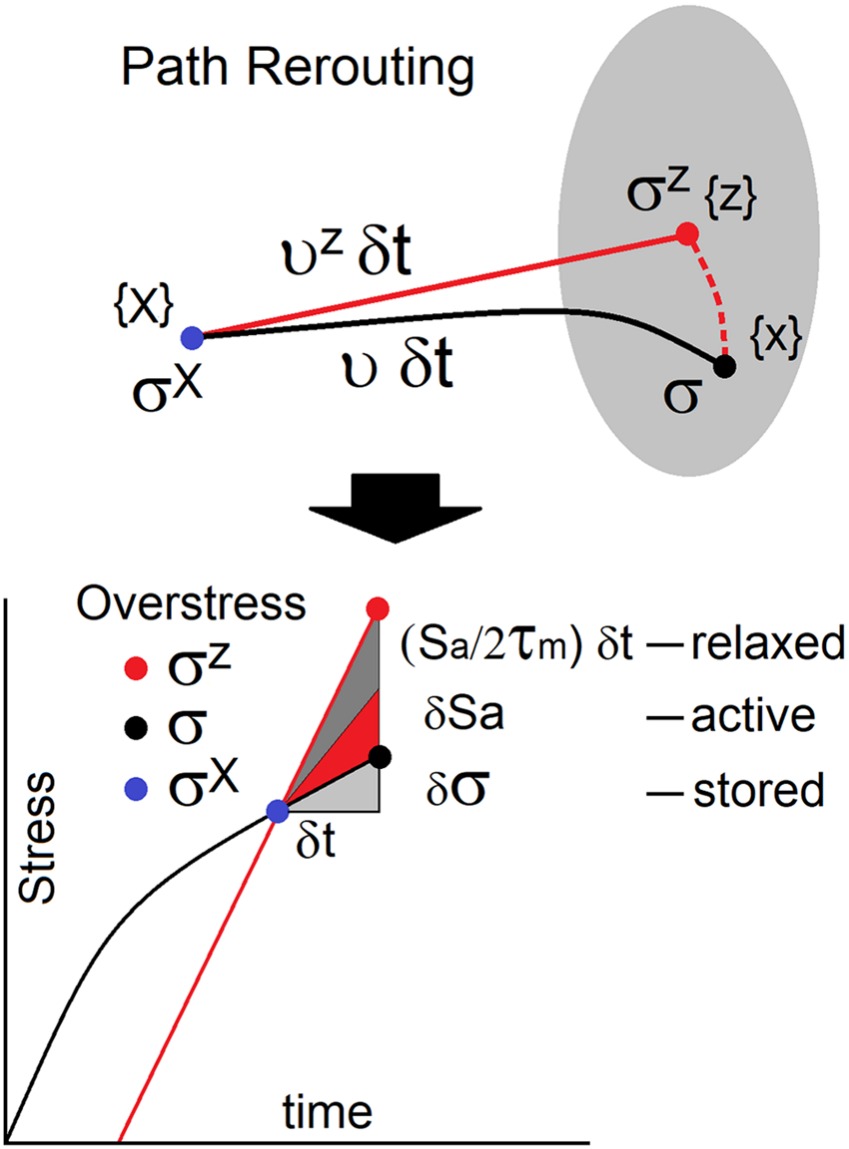
When deformation process is unable to proceed along the previously established trajectory, which would place the material particle in position {z}, then the particle is redirected to a new position {x}. The change produces overstress, which is partly stored and the rest of it triggers dynamic perturbations.

Assume that the previously established deformation pathway is no longer available and, as a result, the deformation process must be rerouted, and then, the readjustments trigger small scale excitations. We visualize the process by monitoring trajectory of a selected material particle and its surroundings^13^, Fig. 2. Initially, the particle resides in position {***X***} and stress is ***σ***^*X*^. Shortly later (*δt*), the particle is forced to change its trajectory and moves to the position {***x***}, where stress is ***σ***. If the original path were to be preserved, the particle would move to the position {***z***} with velocity ***υ***^*z*^ and stress would be ***σ***^*z*^. In all scenarios, the equations of motion $\nabla \cdot {\boldsymbol{\sigma }}=\rho \,\dot{{\boldsymbol{\upsilon }}}$ must be satisfied. The particle acceleration and mass density are denoted as $\dot{{\boldsymbol{\upsilon }}}$ and *ρ*. The process affects tractions projected onto the surface ∂*V*_0_ normal to the direction of the particle velocity ***n***. The difference in tractions $({{\boldsymbol{\sigma }}}^{z}\cdot {\boldsymbol{n}}\ne {\boldsymbol{\sigma }}\cdot {\boldsymbol{n}})$ measured between positions {***z***} and {***x***} triggers stress perturbations $\delta {\boldsymbol{\sigma }}=\frac{{l}_{m}}{{V}_{0}}{\int }_{\partial {V}_{0}}({\boldsymbol{\sigma }}-{{\boldsymbol{\sigma }}}^{z})\cdot ({\boldsymbol{n}}\otimes {\boldsymbol{n}})dS$. The perturbations are present in a spatial domain characterized by the mesoscale length *l*_*m*_. Next, we reduce volume *V*_0_ to material point and the stress perturbations become $\delta {\boldsymbol{\sigma }}={l}_{m}\,\dot{{\boldsymbol{\psi }}}$, where the momentum tensor is ${\boldsymbol{\psi }}=\rho (\delta {\boldsymbol{\upsilon }}\otimes {\boldsymbol{n}}+{\boldsymbol{n}}\otimes \delta {\boldsymbol{\upsilon }})/2$. Note that the reorganizations affect particle acceleration $\delta \dot{{\boldsymbol{\upsilon }}}=(\dot{{\boldsymbol{\upsilon }}}-{\dot{{\boldsymbol{\upsilon }}}}^{z})$. The overstress (***σ***^*z*^ − ***σ***) is partly stored in the newly created dislocation structures, while the remaining part activates dynamic excitations. Given sufficient time, the overstress is relaxed.

### Drag-controlled dislocation glide and overstress

The stress perturbations disturb and, effectively, slow down the plastic flow ${\dot{{\boldsymbol{H}}}}^{t}={\dot{{\boldsymbol{H}}}}^{e}+({\dot{{\boldsymbol{H}}}}^{p}-\delta {\boldsymbol{\sigma }}/\kappa )$, where ${\dot{{\boldsymbol{H}}}}^{t}$ describes the rate of total strain, the elastic strain rate is ${\dot{{\boldsymbol{H}}}}^{e}$, while ${\dot{{\boldsymbol{H}}}}^{p}$ is the rate of drag-free plastic strain. The term *δ****σ***/*κ* represents drag on dislocations and the drag coefficient is *κ*. As always, elastic response ${\boldsymbol{C}}\cdot {\dot{{\boldsymbol{H}}}}^{t}$ is the fundamental thermodynamics process and, therefore, the elastic behavior is preserved at all times, $\dot{{\boldsymbol{\sigma }}}={\boldsymbol{C}}\cdot {\dot{{\boldsymbol{H}}}}^{e}$. In here, elastic matrix is ***C***. Often, only a part of stress ${\boldsymbol{C}}\cdot {\dot{{\boldsymbol{H}}}}^{t}$ can be absorbed by the lattice. The uncompensated stress gives rise to stress perturbations $\dot{{\boldsymbol{\sigma }}}+{R}_{k}^{-2}\delta {\boldsymbol{\sigma }}/{\tau }_{m}={\boldsymbol{C}}\cdot {\dot{{\boldsymbol{H}}}}^{t}$. Note that the stress perturbations describe viscous drag ${R}_{k}^{-2}\delta {\boldsymbol{\sigma }}/{\tau }_{m}=\kappa \,(\delta \dot{{\boldsymbol{\upsilon }}}\otimes {\boldsymbol{n}}+{\boldsymbol{n}}\otimes \delta \dot{{\boldsymbol{\upsilon }}})/{l}_{m}$. The mesoscale relaxation time *τ*_*m*_ = *κ*/*μ* is in the range of sub-microseconds and shear modulus is denoted as *μ*. The resistance to plastic flow *υ*_*r*_ = *l*_*m*_/*τ*_*m*_, when scaled by shear velocity ${\upsilon }_{s}=\sqrt{\mu /\rho }$, quantifies the flow constraints *R*_*k*_ = *υ*_*r*_/*υ*_*s*_. The constraints are directly linked to the energy excess, where the latter activates the plastic flow (see Methods).

In summary, the three relations ${\dot{{\boldsymbol{H}}}}^{t}={\dot{{\boldsymbol{H}}}}^{e}+({\dot{{\boldsymbol{H}}}}^{p}-\delta {\boldsymbol{\sigma }}/\kappa )$, $\dot{{\boldsymbol{\sigma }}}={\boldsymbol{C}}\cdot {\dot{{\boldsymbol{H}}}}^{e}$ and $\dot{{\boldsymbol{\sigma }}}+{R}_{k}^{-2}\delta {\boldsymbol{\sigma }}/{\tau }_{m}={\boldsymbol{C}}\cdot {\dot{{\boldsymbol{H}}}}^{t}$ represent the material model. When eliminating the stress perturbations *δ****σ***, we obtain $\dot{{\boldsymbol{\sigma }}}={\boldsymbol{C}}\cdot ({\dot{{\boldsymbol{H}}}}^{t}-{\dot{{\boldsymbol{H}}}}^{pe})$ and ${\dot{{\boldsymbol{H}}}}^{t}={\dot{{\boldsymbol{H}}}}^{e}+{\dot{{\boldsymbol{H}}}}^{pe}$. At the same time, the perturbations take the form $\delta {\boldsymbol{\sigma }}={\tau }_{m}\,{R}_{k}^{2}\,{\boldsymbol{C}}\cdot {\dot{{\boldsymbol{H}}}}^{pe}$. In here, the flow constraints *R*_*k*_ slow down the plastic flow, affect the overstress and contribute to the excess of energy. A brief description of the viscoplastic model can be found in Methods. In a plastically incompressible material, the rate of effective (true) plastic strain is ${\dot{{\boldsymbol{H}}}}^{pe}={\dot{{\boldsymbol{H}}}}^{p}/(1+2{R}_{k}^{2})$. This expression was derived in ref.^13^. The overstress ${\boldsymbol{C}}\cdot {\dot{{\boldsymbol{H}}}}^{pe}$ in ${\boldsymbol{C}}\cdot ({\dot{{\boldsymbol{H}}}}^{e}+{\dot{{\boldsymbol{H}}}}^{pe})$ undergoes relaxation best described by the Maxwell’s process ${\dot{{\boldsymbol{S}}}}^{a}+{{\boldsymbol{S}}}^{a}/2{\tau }_{m}={\boldsymbol{C}}\cdot {\dot{{\boldsymbol{H}}}}^{pe}$, where ${\dot{{\boldsymbol{S}}}}^{a}$ is the rate of active overstress, Fig. 2. We emphasize that the expression captures the mesoscale relaxation mechanism. It is worth stating that nanoscale relaxation mechanisms are successfully studied in large-scale molecular dynamics simulations^14^.

As stated above, the path rerouting makes plastic flow a kinematically admissible process. The microstructural adaptivity occurs on the expense of the energy excess ${\dot{W}}_{l}={{\boldsymbol{S}}}^{a}:{\dot{{\boldsymbol{S}}}}^{a}/2\mu$. At low strain rates, the energy is dissipated in relaxing dislocation structures. Still, a small fraction of the energy is converted to heat. High strain rates create conditions, where the energy is temporarily retained in the material and affects plastic flow, increases the energy storage and intensifies plasticity-induced heating.

### Source of plasticity-induced heating

Since stress perturbations $\delta {\boldsymbol{\sigma }}={l}_{m}\,\dot{{\boldsymbol{\psi }}}$ are also expressed in terms of overstress $\delta {\boldsymbol{\sigma }}={\tau }_{m}{R}_{k}^{2}({\boldsymbol{C}}\cdot {\dot{{\boldsymbol{H}}}}^{pe})$, we find that the change of momentum $\dot{{\boldsymbol{\psi }}}={R}_{k}{\boldsymbol{C}}\cdot {\dot{{\boldsymbol{H}}}}^{pe}/{\upsilon }_{s}$ is proportional to the rate of the uncompensated stress $({\boldsymbol{C}}\cdot {\dot{{\boldsymbol{H}}}}^{ep})$. According to the Maxwell’s process $({\dot{{\boldsymbol{S}}}}^{a}+{{\boldsymbol{S}}}^{a}/2{\tau }_{m}={\boldsymbol{C}}\cdot {\dot{{\boldsymbol{H}}}}^{pe})$, the dissipated excitations ${\dot{{\boldsymbol{\psi }}}}_{d}={R}_{k}{{\boldsymbol{S}}}^{a}/2{\upsilon }_{s}{\tau }_{m}$ enable relaxation of dislocation structures, while a small portion of ${\dot{{\boldsymbol{\psi }}}}_{d}$ gives rise to phonon vibrations $({\dot{{\boldsymbol{\psi }}}}_{Q}={\xi }_{ef}{\dot{{\boldsymbol{\psi }}}}_{d})$. The factor *ξ*_*ef*_ may vary during an active process, but in here we assume that *ξ*_*ef*_ is a constant and is estimated to be in the range of 0.01. As plastic deformation advances, the heat sources evolve ${{\boldsymbol{\psi }}}_{Q}(t)={{\boldsymbol{\psi }}}_{p}(t)+{\int }_{{t}_{a}}^{t}[{\xi }_{ef}(t,s)\,{R}_{k}{{\boldsymbol{S}}}^{a}(s)/2{\upsilon }_{s}{\tau }_{m}]ds$. According to the Maxwell process, the term ***S***^*a*^/2*τ*_*m*_ can be replaced by $({\boldsymbol{C}}\cdot \,{\dot{{\boldsymbol{H}}}}^{pe}-{\dot{{\boldsymbol{S}}}}^{a})$, where the overstress ${\dot{{\boldsymbol{S}}}}^{a}$ is associated with phonon vibrations. Therefore, at the mesoscopic scale ${\dot{{\boldsymbol{S}}}}^{a}$ is vanishing quantity. In this expression, momentum ***ψ***_*p*_ is associated with stress required for rerouting the deformation pathways. In here, time *t*_*a*_ specifies the moment at which plastic process is initiated. Heat is produced when the excitations $\dot{{\boldsymbol{\psi }}}$ act on the existing sources of heat ***ψ***_*Q*_ such that $\dot{Q}={{\boldsymbol{\psi }}}_{Q}:\dot{{\boldsymbol{\psi }}}/\rho$. In a plastically incompressible material, the plasticity-induced heat is generated with rate

1 $$\dot{Q}=2{R}_{k}[{R}_{k}\,{\xi }_{ef}{\boldsymbol{C}}\cdot {{\boldsymbol{H}}}^{pe}+{\upsilon }_{s}{{\boldsymbol{\psi }}}_{p}]:{\dot{{\boldsymbol{H}}}}^{pe}.$$

The rate of plasticity-induced heating is proportional to the rate of plastic strain ${\dot{{\boldsymbol{H}}}}^{pe}$, is magnified by plastic deformation ***H***^*pe*^, and is affected by the resistance to flow *R*_*k*_. In the case of plastically isotropic materials, the relation (1) is further simplified $\dot{Q}=2{R}_{k}[{R}_{k}\,{\xi }_{ef}\mu \,{e}^{pe}+{\sigma }_{r}]{\dot{e}}^{pe}$, where the equivalent plastic strain *e*^*pe*^ is a scalar and *σ*_*r*_ is the flow stress (see Methods). The Taylor-Quinney coefficient ${\beta }_{1}=\dot{Q}/{\dot{W}}^{p}$ becomes ${\beta }_{1}=2{R}_{k}[{R}_{k}{\xi }_{ef}\mu \,{e}^{pe}+{\sigma }_{r}]/{\sigma }_{eq}$, where plastic power $\,{\dot{W}}^{p}={\sigma }_{eq}\,{\dot{e}}^{pe}$ is defined in terms of equivalent stress and the rate of equivalent plastic strain.

The most relevant conclusion is that $\dot{Q}$ is explicitly affected by plastic deformation, but is not a part of plastic work.

## Discussion

**Figure 3:**
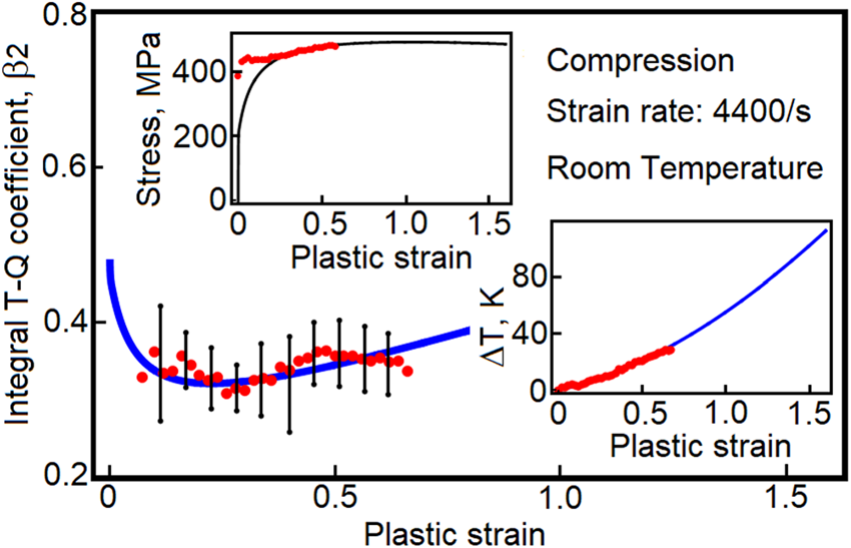
Plasticity-induced heat is calculated for as-received OFHC copper. Red dots represent experimental measurements reproduced from ref.^15^. The error bars suggest large sample-to-sample scatter.

In our analysis, the Taylor-Quinney coefficients *β*_1_ and *β*_2_ are not equivalent because both vary with temperature and plastic strain. The coefficients have been evaluated for many metals and alloys. The issue is that the measurements produce notoriously unreliable data^5,7^. The decision made here is to calibrate the theory for OFHC copper. Samples of as-received copper^15^ have been tested at strain rate 4400/s and the data consists of axial stress, temperature and coefficient *β*_2_; all the quantities are presented in terms of plastic strain. The results provide sufficient information for the calibration of *ξ*_*ef*_ and *σ*_*r*_. The values are ${\xi }_{ef}=9.3\cdot {10}^{-3}$ and *σ*_*r*_ = 105 *Mpa*, respectively. The predictions are presented in Fig. 3. The error bars indicate strong sample-to-sample variability. In the next step, the parameters are recalibrated for annealed OFHC copper, which is one of the most methodically characterized metals. The factor *ξ*_*ef*_ is kept unchanged and stress *σ*_*r*_ is recalibrated such that the same temperature change is obtained in both of the metals. The new value of *σ*_*r*_ is 70 *MPa*.

**Figure 4:**
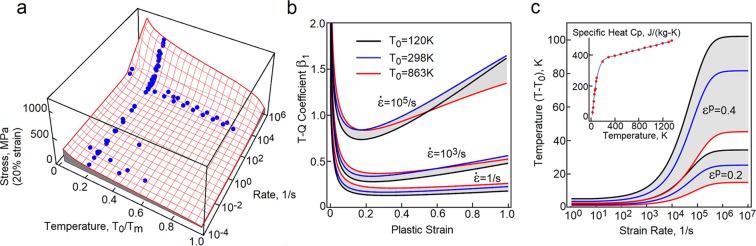
The proposed concept is implemented into a constitutive model for annealed OFHC copper. (**a**) Stress at 20 percent strain (red mesh) is plotted as a function of strain rate and temperature. The blue dots represent experimental data collected from several sources refs^16–23^. (**b**) The instantaneous Taylor-Quinney coefficient is plotted at three strain rates and three temperatures. (**c**) The temperature rise is plotted at plastic strains 20 and 40 percent and at ambient temperatures 120 K, 298 K and 863 K. Specific heat is a temperature dependent variable.

Subsequently, the overstress concept and the description of plasticity-induced heat are incorporated into our constitutive model for copper. The model correctly predict stress-strain responses at strain rates from diffusional flow to extreme dynamics and temperatures from cryogenic to nearly melting point^13^. Quality of the predictions is demonstrated in Fig. 4a, where the plot of stress at 20% strain (red mesh) is constructed in terms of strain rate and temperature. The data points (blue dots) are collected from multiple sources^16–23^. In Fig. 4b,c, plasticity-induced heat is computed at three temperatures. The black lines capture trends at 120 K, the blue ones depict the room temperature responses and the red lines represent the behaviors at 863 K. Coincidentally, temperature 120 K is recorded outside the International Space Station and 863 K is estimated to be the average temperature on the Venus surface.

### Low strain rates (up to 100/s)

**Figure 5:**
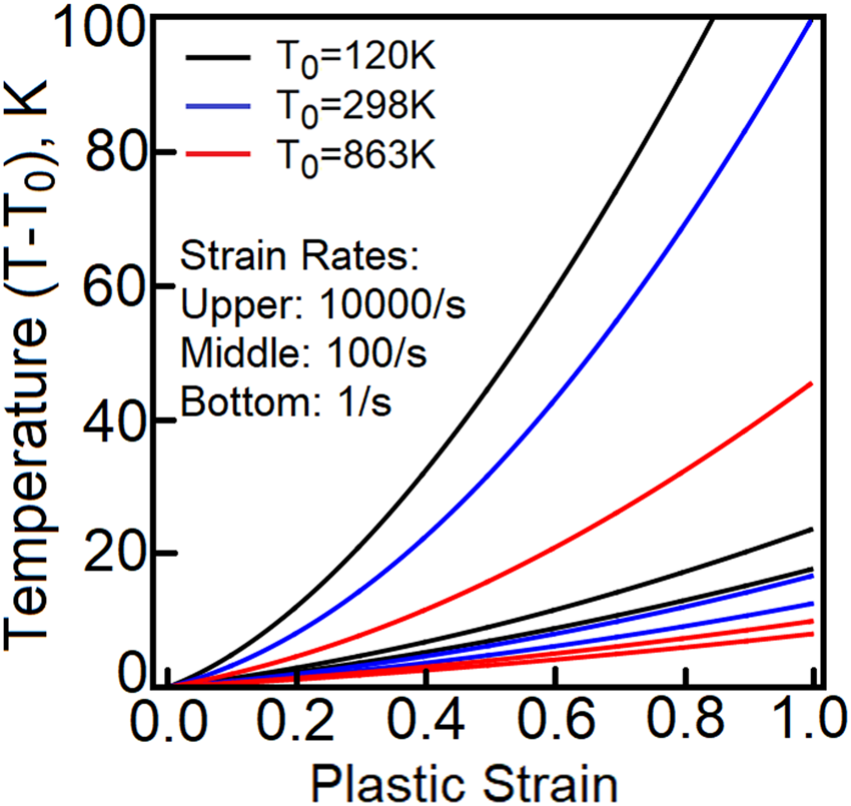
The change of temperature is plotted as a function of plastic strain for three temperatures 120 k, 298 K and 863 K and three strain rates (1/s, 100/s and 10000/s).

It should be stated that the change of temperature (and not the T-Q coefficient) is an experimentally measured quantity. At low rates, the thermomechanical coupling further complicates the evaluations. It is reported that strain rates not exceeding 100/s make the Taylor-Quinney coefficient mildly sensitive to the rate of loading and the sensitivity becomes negligible at quasi-static conditions. It has been shown^24^ that low strain rate in polycrystalline OFHC copper may affect the average Taylor-Quinney coefficient, but the differences are not significant. The results obtained on Al-1% Mn alloy^25^ subjected to strain rates 0.25/s, 2.5/s and 25/s further confirm the observation. Such trends are also reported in ref.^26^, where 2024 aluminum alloy is subjected to strain rates 0.1/s up to 10/s and steel has been tested at strain rates between 0.25/s and 25/s. This type of behavior has been confirmed in calculations performed on Fe-30% Ni Austenite^27^ at three temperatures (1123 K, 1223 K and 1323 K) and three strain rates (0.1/s, 1/s and 10/s). Our predictions properly reproduce the observed trends, Fig. 5, where the temperature rise is plotted as a function of plastic strain at three strain rates (10000/s, 100/s and 1/s) and three temperatures (120 K, 298 K and 863 K). The strain rate sensitivity is predicted to be an irrelevant factor at strain rates below 50/s. Our understanding is that elevated temperatures and low strain rates soften the path-rerouting constraints, thus, only a small amount of energy is converted to heat.

### High strain rates (above 1000/s)

At high strain rates, the Taylor-Quinney coefficient becomes sensitive to the rate of loading. The predictions match the trends reported in refs^9,28^. As stated in ref.^29^, the change of temperature rises faster at lower temperatures. Note that the concepts is capable of capturing the trends at low and high strain rates, Figs 4c and 5. In Fig. 4c, the change of temperature is calculated at plastic strains 20 and 40 percent and is plotted as a function of strain rate.

### Extreme strain rates (above 10^5^/s)

**Figure 6:**
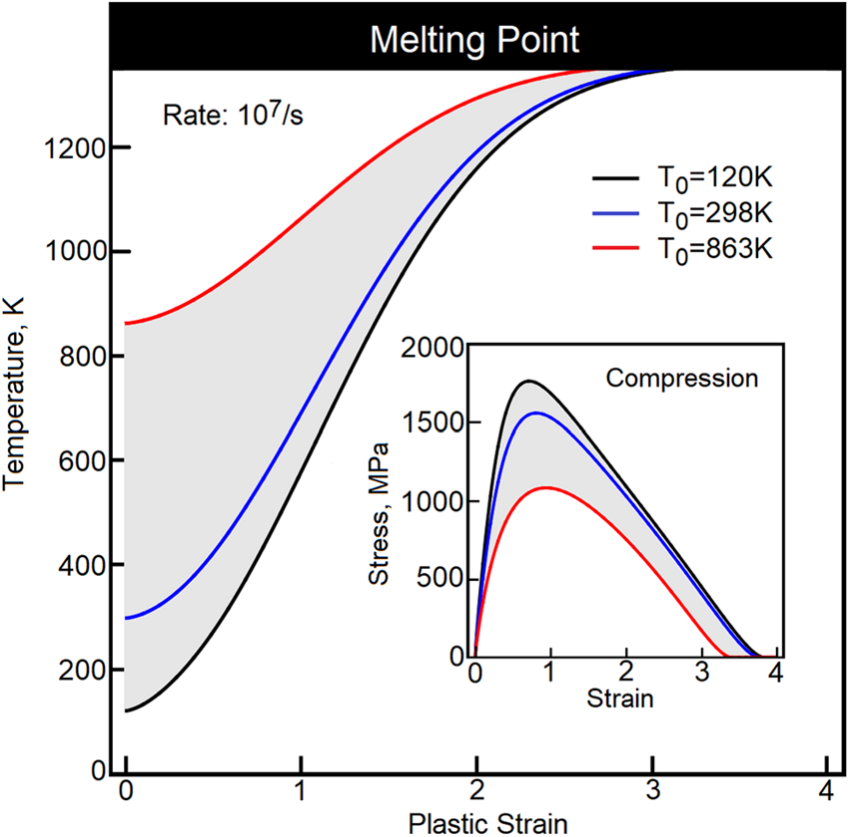
The plasticity-induced change of temperature is plotted as a function of plastic strain at strain rate 10^7^/*s*. Ambient temperatures are 120 K, 298 K and 863 K.

In Fig. 6, a plot of the temperature rise is constructed for annealed OFHC copper subjected to compression at the strain rate 10^7^⁄s and strains up to 400%. The plot is generated at temperatures 120 K, 298 K and 863 K. At extreme rates, we expect the rate of plasticity-induced heat to slow down, Fig. 6. Our interpretation is that the high-strain-rate resistance to plastic flow *R*_*k*_ is already fully maximized (*R*_*k*_ → 1). At this stage of deformation, other mechanisms might be at play. Among such mechanisms are crystallographic reorientations, recrystallizations, etc.

Plasticity-induced heating is intensely studied on samples, where adiabatic shear bands dominate the material’s behavior^30,31^. One may envision that a favorable arrangement of defects creates conditions for a runaway instability, where localized shear magnifies plasticity-induced heating, strain rate is intensified and all the factors close the runaway loop. It seems quite evident that large shear strains, when executed quickly, can bring the material to melting point. For example, partial melting is reported under the conditions of dynamic punching of aluminum tubes^32^. Thus, the melting regime requires very large deformation applied with extreme strain rates^33^. In another case, tests conducted on Al-Sc alloy^34^ indicate that strain rates up to 6.2 10^5^/*s* and strains larger than 400 percent do not cause melting and, instead, the material exhibits increased microhardness. Much higher rates can be accomplished in explosively driven experiments^35^, where strain rates in shear bands are estimated to be about 10^8^/*s*.

### Final remarks

Throughout the twentieth century, we were accustomed to the interpretation credited to Taylor and Quinney. In this paper, we suggest that the problem of plasticity-induced heat is far from understood. As Jim Langer stated^11^, “*much of dislocation theory as practiced today is based on questionable and sometimes demonstrably incorrect phenomenological assumptions. As a result, there are many technologically important behaviors that we have not understood and which need now to be restudied*”. His arguments are compelling, stretch much beyond the scope of the study, and should be carefully examined. Unquestionably, molecular dynamics simulations offer an invaluable insight into the small-scale-plasticity phenomena. Nowadays, the most sophisticated calculations can be performed on samples that are not larger than one cubic micrometer^14^. For this reason, these studies are incapable of reproducing the mesoscopic complexities, which in metals seem to be of a paramount importance. Realistic simulations might require atomistic samples that are one cubic millimeter or larger. Such an analysis is simply not achievable. For this reason, continuum-level plasticity will remain the engineering choice for a foreseeable future.

## Methods: Mechanisms-Based Constitutive Model

The concept is integrated into our viscoplasticity model. The description is summarized in five subsections.

1. The Weibull-based thermal activation factor is ${A}_{D}(T)=1-exp[-{g}_{a}{(\frac{{T}_{c}}{T}-\frac{{T}_{c}}{{T}_{m}})}^{{k}_{a}}]$. In this expression, a transition of flow mechanism occurs at *T*_*c*_ and melting point is *T*_*m*_. The rate of dislocation multiplication *k*_*a*_ further quantifies thermal activation. The factor *g*_*a*_ is a constant.
2. In elastically isotropic metals, the rate of effective plastic strain is ${\dot{{\boldsymbol{H}}}}^{pe}={{\boldsymbol{N}}}^{{\boldsymbol{\sigma }}}\,{\dot{e}}^{pe}/2$, where tensor ***N***^***σ***^ = ***N***_1_ − ***N***_3_ describes the Tresca slip mechanism. In here, first and third principal stresses are *σ*_1_ = ***N***_1_:***σ*** and *σ*_3_ = ***N***_3_:***σ***. The rate of equivalent plastic strain is denoted as ${\dot{e}}^{pe}$.
3. The viscoplasticity model couples the rate of equivalent plastic strain ${\dot{e}}^{pe}$ with equivalent stress *σ*_*eq*_ (maximum shear stress)${\dot{e}}^{pe}={\dot{{\rm{\Lambda }}}}_{p}\frac{M\,{{\rm{\Lambda }}}_{0}}{1+2{R}_{k}^{2}}{({\sigma }_{eq}/{\sigma }_{0}^{p})}^{{n}_{p}}$, where yield stress is ${\sigma }_{0}^{p}={\sigma }_{0}{A}_{D}$ and *σ*_0_ is a constant. The stress exponent *n*_*p*_ determines the elastic-plastic transition, while the strain rate sensitivity is tuned by the rate factor ${\dot{{\rm{\Lambda }}}}_{p}$. The average Schmid factor *M* controls plastic hardening. In this model, the factor specifies misorientation of slip planes. For this reason, *M* is a function of plastic strain, temperature and the resistance to flow, *M* = *M*(*e*^*pe*^, *A*_*D*_, *R*_*k*_).
4. The active overstress ***S***^*a*^ determines the energy excess ${\dot{W}}_{l}={{\boldsymbol{S}}}^{a}:{\dot{{\boldsymbol{S}}}}^{a}/2\mu$. Energy *W*_*l*_ contributes to the development of flow constraints such that ${R}_{k}=1-{R}_{k}^{0}exp[-{({W}_{l}/{G}_{R})}^{{k}_{R}}]$. We estimate that the parameter to be ${R}_{k}^{0}=0.89$ and, therefore the initial resistance to flow is 0.11, while G_R_ and k_R_ are constants.
5. The rate of heat $\dot{Q}=2{R}_{k}[{R}_{k}\,{\xi }_{ef}\,{\boldsymbol{C}}\cdot {{\boldsymbol{H}}}^{pe}+{\upsilon }_{s}{{\boldsymbol{\psi }}}_{p}]:{\dot{{\boldsymbol{H}}}}^{pe}$ is further simplified. Since ${\dot{{\boldsymbol{H}}}}^{pe}={{\boldsymbol{N}}}^{{\boldsymbol{\sigma }}}{\dot{e}}^{pe}/2$, we have $\dot{Q}=2{R}_{k}[{R}_{k}\,{\xi }_{ef}\mu \cdot {e}^{pe}+{\upsilon }_{s}{{\boldsymbol{N}}}^{{\boldsymbol{\sigma }}}:{{\boldsymbol{\psi }}}_{p}/2]{\dot{e}}^{pe}$, where the term $({\boldsymbol{C}}\cdot {{\boldsymbol{H}}}^{pe}):{{\boldsymbol{N}}}^{{\boldsymbol{\sigma }}}/2$ is further approximated and becomes *μe*^*pe*^. The assumption is that the factor *ξ*_*ef*_ absorbs any discrepancy that result from the approximation. The flow stress is ${\sigma }_{r}={\upsilon }_{s}\,{{\boldsymbol{N}}}^{{\boldsymbol{\sigma }}}:{{\boldsymbol{\psi }}}_{p}/2$ and we obtain $\dot{Q}=2{R}_{k}[{R}_{k}\,{\xi }_{ef}\mu \,{e}^{ep}+{\sigma }_{r}]{\dot{e}}^{pe}$.

Complete description of the model can be found in ref.^13^.
